# Regarding the Charmed-Strange Member of the 2^3^ *S*_1_ Meson State

**DOI:** 10.1155/2013/658105

**Published:** 2013-10-22

**Authors:** Xue-Chao Feng, Jing Chen

**Affiliations:** Department of Technology and Physics, Zhengzhou University of Light Industry, Zhengzhou 450002, China

## Abstract

By employing the mass relations derived from the mass matrix and Regge trajectory, we investigate the masses of charmed and charmed-strange members of the 2^3^ *S*_1_ meson. The masses are compared with the values predicted by other theoretical approaches and experimental data. The results may be useful for the discovery of the unobserved meson and the determination of the quantum number of the newly discovered states.

## 1. Introduction

 Charmed spectroscopy becomes an active field with many new states observed in the experiment in the last few years [1–8]. The *D*_*SJ*_(2632) (with the mass 2632.5 ± 1.7 MeV and decay width Γ_tot_ ≤ 17 MeV) was reported in the two decay modes *D*_*s*_^+^ *η* and *D* ^0^ *K* ^+^ by the Selex collaboration [5]. In [9–11], *D*_*SJ*_(2632) was interpreted as the first radical excitation of *D*_S_*(2112). However, the narrow and dominated *D*_*s*_^+^ *η* decay mode implies that the assignment is problematic. On the other hand, the mass of the *D*_*SJ*_(2632) is lower than the value of the tradition potential model predictions. Therefore, the *D*_*SJ*_(2632) is also interpreted as a tetraquark, a hybrid, a diquark-antiquark bound state, and so forth.

Recently, Belle collaboration observed a new $c\overset{-}{s}$ state *D*_*SJ*_(2700) with a mass of 2708 ± 9_−10_^+11^ MeV and width 108 ± 23_−31_^+36^ MeV [8]. Based on its observed decay channel $B^{+}\to {\overset{-}{D}}^{0}D_{SJ}\to {\overset{-}{D}}^{0}D^{0}K^{+}$, the *D*_*SJ*_(2700) resonance is interpreted as a charmed-strange meson state.

Firstly, we reviewed the assignment of the 2^3^ *S*_1_ meson state in the $q\overset{-}{q}$ quark model (see Table 1). According to the new edition of Particle Data Group (PDG) [12], the states *ω*(1420) and *ϕ*(1680) have been well established as the isoscalar member in the 2^3^ *S*_1_ meson state. K*(1410) is assigned as isodoublet member however, it is still based on very weak experimental signals. Till now, the K*(1410) has been reported by two experiments *K* ^−^ *p* → *K* ^−^ *π* ^+^ *n* (with mass 1380 ± 21 ± 19 MeV and width 176 ± 52 ± 22 MeV) and $K^{-}p\to {\overset{-}{K}}^{0}\pi^{+}\pi^{-}n$ [12] (with mass 1420 ± 7 ± 10 MeV and width 240 ± 18 ± 12 MeV). The assignment of isodoublet of the 2^3^ *S*_1_ meson state was investigated in our previous work [13]. For the heavy-light meson, the charmed-strange member of the 2^3^ *S*_1_ meson state has attracted more attention, recently. Both the *D*_*SJ*_(2632) and the *D*_*SJ*_(2700) are probably interpreted as charmed-strange candidate of the 2^3^ *S*_1_ meson state [9–11, 14]. The nonstrange partner of *D*_*SJ*_ has not been observed in the experiment.

In this work, based on the isoscalar states can mix to form the physical states in the $q\overset{-}{q}$ quark model and the linear Regge trajectory; we establish the new mass relations which relate the mass spectrum of meson state and constituent quark masses. Inserting the corresponding constituent quark masses and the following well-established states, we reexamine the mass spectrum of the 2^3^ *S*_1_ meson state. The results could be a useful comparison with the experiment data in the new experiment.

## 2. Mass Matrix and Regge Trajectory

In the quark model, the two isoscalar states with the same *J* ^*PC*^ will mix to form the physical isoscalar states. We can establish the mass-squared matrix in the $s\overset{-}{s}$ and $N=(u\overset{-}{u}\;+\;d\overset{-}{d})/\sqrt{2}$ basis [25] as follows:

(1) M2=(Mρ(1450)2+2Ann2Ans2Ans2MK∗(1410)2−Mρ(1450)2+Ass),

where *M*_*ρ*(1450)_ and *M*_K*(1410)_ are the masses of isovector and isodoublet states of the 2^3^ *S*_1_ meson nonet, respectively and *A*_*nn*_, *A*_*ns*_, and *A*_*ss*_ are the mixing parameters which describe the $q\overset{-}{q}↔q^{\prime }\overset{-}{q^{\prime }}$ transition amplitudes. In order to reduce the number of parameters, we adopt the similar expression of the transition amplitudes in the $q\overset{-}{q}↔q^{\prime }\overset{-}{q^{\prime }}$ process which is widely used in [26–28] as follows:

(2) Ann=Λmnmn,Ass=Λmsms,  Ans=Λmnms,

where Λ is a phenomenological parameter. Based on the isospin symmetry, we have $m_{u}=m_{\overset{-}{u}}=m_{d}=m_{\overset{-}{u}}$, $m_{s}=m_{\overset{-}{s}}$ (*m*_*n*_, *m*_*s*_ denote the mass of light quark *u*, *d*, and *s*).

In the 2^3^ *S*_1_ meson nonet, we assume that the physical states *ω*(1420) and *ϕ*(1680) are the eigenstates of mass-squared matrix and the masses square of *M*_*ω*(1420)_^2^ and *M*_*ϕ*(1680)_^2^ are the eigenvalues, respectively. The physical states *ω*(1420) and *ϕ*(1680) can be related to the $s\overset{-}{s}$ and $N=(u\overset{-}{u}+d\overset{-}{d})/\sqrt{2}$ by

(3) (|ω(1420)〉|ϕ(1680)〉)=U(|N〉|S〉),

and the unitary matrix *U* can be described as

(4) UM2U†=(Mω(1420)200Mϕ(1680)2).

According to relations (1), (2), (3), and (4), we will obtain

(5) 2Λmn2+2MK∗(1410)2+Λms2=Mω(1420)2+Mϕ(1680)2,(Mρ(1450)2+2Λmn2)(2MK∗(1410)2−Mρ(1450)2+Λms2)   −2Λms2mn2=Mω(1420)2∗Mϕ(1680)2.

In relation (5), the masses of K*(1410) and *ϕ*(1680) are related with constituent quarks mass and phenomenological parameter Λ.

Regge theory is cornered with the particle spectrum, the forces between particles, and the high energy behavior of scattering amplitudes. Because the Regge trajectories can offer an effective way for the assignment and the classification of meson states, it also become an active field with many new particles and resonances being observed in the experiment in the last decade. In [29], the authors investigated the mass of different meson multiplets and suggested that the quasilinear Regge trajectories could describe the meson mass spectrum. Khruschov [30], using the phenomenology formulae deprive from the Regge trajectories, predicted the masses of excited meson states. Anisovich et al. [31] show that meson states can fit to the quasi-linear Regge trajectories with good accuracy.

According to the hadron with a set of given quantum numbers belonging to a quasilinear trajectory, we will have the following relation [7]:

(6) J=αii′−(0)+αii′−′Mii′−2,

where $i\overset{-}{i^{\prime }}$ refers to the quark (antiquark) flavor, *J* and $M_{i\overset{-}{i^{\prime }}}$ are, respectively, the spin and mass of the $i\overset{-}{i^{\prime }}$ meson. The parameters $\alpha_{i\overset{-}{i^{\prime }}}^{\prime }$ and $\alpha_{i\overset{-}{i^{\prime }}}(0)$ are, respectively, the slope and intercept of the trajectory. The intercepts and slopes can be described by [15, 29]

(7) αii−(0)+αjj−(0)=2αij−(0),

(8) 1αii′−′+1αjj′−′=2αij′−′.

Relation (7) is satisfied in two-dimensional QCD [32], the dual-analytic model [33], and the quark bremsstrahlung model [34]. Relation (8) is derived from the topological and the $q\overset{-}{q}$-string picture of hadrons [35]. According to available data of meson states, Burakovsky constructed a slope formula (9) for all quarks flavors [36] follows:

(9) π4αji′+π4α′mi+mj2αji′=α′,

where *m*_*i*_ and *m*_*j*_ are the corresponding constituent quark masses and the *α*′ = 0.88 GeV^−2^ is the standard Regge slope in the light quark sector.

From relations (6)–(9), we obtained the following relation:

(10) Mρ(1450)2α′2+2α′mn+Mψ(2S)2α′2+2α′mc=2MD2α′2+α′(mn+mc),4MK∗(1410)2α′2+α′(ms+mn)+Mψ(2S)2α′1+α′mc  =Mρ(1450)2α′2+α′mn+4MDs2α′2+α′(ms+mc).

Using relations (5) and (10), we can obtain the masses of K*(1410), *D*, and *D*_*s*_ in the 2^3^ *S*_1_ meson state. In this paper, we use the average values of the constituent quark mass as input parameters (see Table 2). The mass of *ω*(1420), *ϕ*(1680), and *ψ*(2*S*) used are taken from the new edition of PDG [1]. Our results are presented in Table 3. 

If *M*_K*(1410)_ = 1579.82 MeV and *M*_*ω*(1420)_ and *M*_*ϕ*(1680)_ are assigned as the member of the 2^3^ *S*_1_ meson nonet, we can investigate the quarkonia content of isoscalar state. Based on the previous assumption that physical states *ω*(1420) and *ϕ*(1680) are the eigenvectors of mass-squared matrix, the unitary matrix *U* can be described as

(11) U=(Xω(1420)Yω(1420)Xϕ(1680)Yϕ(1680)).

Inserting the masses of isoscalar states, we obtain the quarkonia content of *ω*(1420) and *ϕ*(1680) as follows:

(12) |ω(1420)〉=−0.997|N〉−0.074|S〉,|ϕ(1680)〉=0.074|N〉−0.997|S〉.

## 3. The Results and Conclusions

In summary, based on the mass matrix and Regge trajectory, we investigate the masses of K*(1410), *D*, and *D*_*s*_ in the 2^3^ *S*_1_ meson state. The mass of K*(1410) is determined to be 1579.82 MeV; the results is consistent with our previous work. For the heavy-light meson section, we predicted the mass of charmed-strange member to be 2700 MeV. The value is larger than *D*_*SJ*_^+^(2632) and in agreement with the *D*_*SJ*_(2700). Moreover, on the basis of unusual decay modes and narrow width of the *D*_*SJ*_^+^(2632), we suggest that the *D*_*SJ*_(2700) should be the first radial excitation of the *D*_S_*(2112). As a byproduct, the quarkonia content of *ω*(1420) and *ϕ*(1680) was offered, which implies that *ω*(1420) is pure $u\overset{-}{u}+d\overset{-}{d}$ state and *ϕ*(1680) is pure $s\overset{-}{s}$ state. Our results should be useful for the assignment and the identification of the member of the 2^3^ *S*_1_ meson state in the new experiment.

## Figures and Tables

**Table 1:** Assignment of the 2^3^ *S*_1_ meson state in PDG [12].

^2*S*+1^ *L*_*J*_	$n\overset{-}{n}$	$n\overset{-}{n}(s\overset{-}{s})$	$n\overset{-}{s}$	$c\overset{-}{c}$	$c\overset{-}{n}$	$c\overset{-}{s}$
2^3^ *S*_1_	*ρ*(1450)	*ω*(1420) *ϕ*(1680)	K*(1410)	*ψ*(2*S*)	*D*	*D*_*s*_

**Table 2:** Constituent quark masses (in MeV) in different phenological models.

Mass	Ref [15]	Ref [16]	Ref [17]	Ref [18]	Ref [19]	Average
*m*_*n*_ (*n* = *u*, *d*)	290	360	337.5	311	310	321
*m*_*s*_	460	540	486	487	483	491.2
*m*_*c*_	1650	1710	1550	1592		1625.5

**Table 3:** Masses (MeV) of K*(1410), *ϕ*(1680), and *ψ*(2*S*) in the 2^3^ *S*_1_ meson state.

Mass	*M*_K*(1410)_	*M*_*D*_	*M*_*D*_*s*__
Our results	1579.82	2593.2	2700
Ref [12]	1414 ± 15		
Ref [14]	1567 ± 22.6		
Ref [20]	1573	2588	
Ref [21]			2757.8
Ref [22]		2629	2716
Ref [23]		2640	2730
Ref [24]		2620	2730
